# Risk Factors of Internet Addiction among Internet Users: An Online Questionnaire Survey

**DOI:** 10.1371/journal.pone.0137506

**Published:** 2015-10-13

**Authors:** Chia-Yi Wu, Ming-Been Lee, Shih-Cheng Liao, Li-Ren Chang

**Affiliations:** 1 School of Nursing, National Taiwan University College of Medicine, Taipei, Taiwan; 2 Taiwan Suicide Prevention Center, Taipei, Taiwan; 3 Department of Psychiatry, National Taiwan University College of Medicine, Taipei, Taiwan; 4 Department of Psychiatry, National Taiwan University Hospital, Taipei, Taiwan; Univ of Toledo, UNITED STATES

## Abstract

**Backgrounds:**

Internet addiction (IA) has become a major public health issue worldwide and is closely linked to psychiatric disorders and suicide. The present study aimed to investigate the prevalence of IA and its associated psychosocial and psychopathological determinants among internet users across different age groups.

**Methods:**

The study was a cross-sectional survey initiated by the Taiwan Suicide Prevention Center. The participants were recruited from the general public who responded to the online questionnaire. They completed a series of self-reported measures, including Chen Internet Addiction Scale-revised (CIAS-R), Five-item Brief Symptom Rating Scale (BSRS-5), Maudsley Personality Inventory (MPI), and questions about suicide and internet use habits.

**Results:**

We enrolled 1100 respondents with a preponderance of female subjects (85.8%). Based on an optimal cutoff for CIAS-R (67/68), the prevalence rate of IA was 10.6%. People with higher scores of CIAS-R were characterized as: male, single, students, high neuroticism, life impairment due to internet use, time for internet use, online gaming, presence of psychiatric morbidity, recent suicide ideation and past suicide attempts. Multiple regression on IA showed that age, gender, neuroticism, life impairment, internet use time, and BSRS-5 score accounted for 31% of variance for CIAS-R score. Further, logistic regression showed that neuroticism, life impairment and internet use time were three main predictors for IA. Compared to those without IA, the internet addicts had higher rates of psychiatric morbidity (65.0%), suicide ideation in a week (47.0%), lifetime suicide attempts (23.1%), and suicide attempt in a year (5.1%).

**Conclusion:**

Neurotic personality traits, psychopathology, time for internet use and its subsequent life impairment were important predictors for IA. Individuals with IA may have higher rates of psychiatric morbidity and suicide risks. The findings provide important information for further investigation and prevention of IA.

## Introduction

Rapidly increasing use of smart phones, tablets, and computers has made internet an indispensable part in modern society. The negative impact of excessive, maladaptive or addictive internet use has attracted much research attention. In particular, internet addiction (IA) has become a major public health issue worldwide and brought about a dramatic proliferation of research in this area [1–7]. IA is defined as a pathological pattern of internet use, which is also described as internet dependence, compulsive internet use, problematic internet use, internet abuse, and pathological internet use [8]. The user cannot self-control the use of internet, resulting in significant impairments at school, home, work, health or interpersonal relationships [1]. They may find it difficult to stop using the internet due to its anonymity, convenience and accessibility and may use it as a way to escape reality [9]. The types of activity involved in IA include online gaming, social networking, online gambling, online shopping, virtual sex and information overload.

Internet addiction in young people is especially recognized as a social problem. Previous epidemiological studies using community self-report survey reported that the prevalence of IA in adolescents ranged from 38% to 90% [2, 3, 10–19]. Reasons for a wide range of prevalence rates may be related to different study designs, diverse assessment measures, or various diagnostic criteria, cultural backgrounds, and study samples. The conceptualization and definition of IA as a specific independent psychiatric disorder are still in debate by health professionals. One of the central criticisms is that IA was found to be highly co-morbid with other mental health conditions, such as attention-deficit hyperactivity disorder (ADHD) [20–22], depression [23], anxiety disorders [21], low self-esteem [24], impulsivity [25], social anxiety [26], and suicide. [27–28]. Moreover, some researchers have proposed to focus on addiction to particular internet “activity" rather than “internet” per se. For example, the American Psychiatric Association (APA) proposed the “Internet Gaming Disorder” in 2013 as a health condition for further study [29]. There is room for future studies of IA in order to build further evidence to inform the prevention and treatment of IA. However, few studies have considered personality factor concurrently in multiple regression analysis, especially personal trait of anxiety that may correlate with frequent internet use [21]. Most previous studies on IA centered on the young population, particularly the adolescents [7]. But internet use has made a great and wide influence on the society for different age groups. Therefore, the present study aimed to investigate the prevalence of IA and its psychosocial determinants as well as co-morbid psychiatric morbidities among internet users across different age groups.

## Materials and Methods

### Participants

The present study was a cross-sectional online questionnaire survey performed during February 1st to April 30th, 2014. The convenient sample was recruited through public announcement at the Taiwan Suicide Prevention Center website that contains an invitation of participating in an "Internet Use and Health" survey. The study was ethically approved by the National Taiwan University Hospital (201204034RIC). Informed consent was acquired before each participant administered the questionnaire online. Therefore, potential participants were introduced with the study purpose and contents before entering the study. After they were fully instructed and acknowledged the anonymity and de-linkage principle together with other ethical considerations, they carried on fulfilling the questions online based on their own willingness. The consent was not electronically recorded but all participants agreed to the study aim and contributed in this online survey. The participants were invited to complete a series of psychometric measures as listed below.

### Measurement

#### The CIAS-R

The CIAS-R was developed for assessing the internet use problems, particularly for Chinese populations [30]. It is a self-rating questionnaire comprising 26 items with a four-point Likert's scale ranging from 1 (does not match my experience at all) to 4 (definitely match my experience). A higher score indicates a greater severity of addiction to internet activities. The scale consists of five subscales: 1) compulsive use (5 items); 2) withdrawal symptoms (5 items); 3) tolerance (4 items); 4) interpersonal and health-related problems (7 items); and 5) time management problems (5 items). According to Ko's report, the optimal cutoff point at 67/68 score to determine IA via clinical interview had a good performance on reliability and validity [31,32]. In the current study, we adopted this cutoff value to classify IA.

#### Five-item Brief Symptom Rating Scale (BSRS-5) and personal experience of suicide

The BSRS-5 is a 5-item Likert's scale by self-report for measurement of the severity of psychological distress. A higher score indicates poorer mental health [33]. The full scale contains the following 5 items of psychopathology: 1) having trouble falling asleep (insomnia); 2) feeling tense or keyed up (anxiety); 3) feeling easily annoyed or irritated (hostility); 4) feeling blue (depression);and 5) feeling inferior to others (interpersonal hypersensitivity: inferiority). An additional question, “Do you have any suicide ideation?” was added at the end of the questionnaire.

The subjects were asked to rate the degree of distress caused by each item during the past week, including the current day, on a five-point scale: 0, not at all; 1, a little bit; 2, moderately; 3, quite a bit; 4, extremely; a total score was calculated for each subject. The BSRS-5 has been reported to have satisfactory psychometric properties as a measure to detect psychiatric morbidity and to predict suicide ideation in medical settings or the community [34, 35]. In this study, presence of psychiatric morbidity was defined as BSRS-5 score 6 or greater [33]. In addition, the participants were also asked about if they had any suicide attempt over the past one year or across the lifetime.

#### MPI- Neuroticism Scale (NS) and Social Desirability Scale (SDS)

The NS consists of 13 items of descriptions about personality traits, selected from the Maudsley Personality Inventory (MPI) [36–38]. Each item was rated according to personal experience as "yes,?, or No". "Yes" response indicates the individual agrees to the description suitable for him. The "?" response indicates that the respondents had difficulty to make a clear decision. Neuroticism is characterized by high levels of negative and unstable affect, such as feeling depressed, feeling restless, feeling guilty, and mood swing as well as poor concentration or distractibility. The higher NS score means the higher trends of neuroticism, which was considered to be more prone to poorer mental health. In this study, score of 15 or more (one standard deviation above the mean) are defined as high Neuroticism. Besides, the additional 4 items of social desirability were applied for reference as a lie scale. The subjects with full positive responses for all 4 items were considered as highly unreliable and excluded for analysis. In this study, no subject rated full responses to SDS among the 1100 participants. The Cronbach’s alpha for SDS was .29, which was low because of the limited number items of the scale. In this study, it was used as a lie scale and not analyzed as a risk factor.

#### Demographics and internet use habits

The participants were asked to fill out demographic information including gender, age, education, marital status, residence, and occupation. In addition, they were requested to fulfill the following assessment related to internet use habits: 1) online activities: multiple choices on three categories of gaming, social networking and others (e.g., shopping, gambling, video watching); 2) average internet use time (hours per week) except for school work or job-required tasks; and 3) life impairment due to internet use (i.e., disruption of normal activities on schooling, family, occupation, or interpersonal relationship); rating on four levels of severity for internet gaming disorder specified by DSM5 (i.e., 1 = none; 2 = mild; 3 = moderate; and 4 = severe).

### Statistical analysis

Apart from descriptive statistics of demographic and psychosocial correlates of IA, we performed the t-test or F-test to examine the CIAS-R scores in different groups of independent variables and Pearson’s correlation to examine the associations between CIAS-R and other correlates. Cronbach’s alpha was used to estimate the internal consistency of CIAS-R, BSRS-5, and NS. Moreover, we used multiple regression to determine independent psychosocial predictors for IA scores. Path analysis of structural equation model was performed to demonstrate the structural relationships among those significant risk factors and IA score. Furthermore, we examined the associations between IA (CIAS-R score) and psychopathology, neuroticism and other psychosocial variables by using logistic regression analyses with estimation of odds ratios and 95% confidence intervals. Multivariate logistic regression analysis was performed to identify key predictors of the IA after adjustment for age and gender. Statistical significance was set at a level of p < .05. The SPSS 19.0 software package (SPSS, Chicago, IL) was used for analyses in this study.

## Results

### Demographic and psychosocial information

The sample size recruited online was 1100 subjects. As S1 Table showed, the study participants comprised 156 males (14.2%) and 944 females (85.8%). The majority people (67.5%) were at the age of 25–44 years, with 93.3% above the education level of high school. The subjects’ residential areas were distributed across Taiwan, including New Taipei City (19.7%), Tainan (15.6%), Taipei (14.1%), Kaohsiung (11.6%), Taichung (11.0%), and other areas (27.9%). The distribution is representative of the population size in different cities of Taiwan. Approximately eighty percent of the subjects were employed. Besides, the main reasons for internet use included social networking (86.7%), gaming (23.6%) and others (43.0%).

### Prevalence of internet addiction and associated psycho-social risk factors

The internal consistency (i.e., the Cronbach’s alphas) of main scales of this study was satisfactory: .96 for CIAS-R, .89 for BSRS-5, and .91 for NS. Based on reliable measurements, the individuals with the following characteristics were found to present with significantly higher scores of CIAS-R (S1 Table): male gender, single, students, online gaming, higher amount of time spent online (13 hours or more per week), higher N score (15 and higher), presence of psychiatric morbidity, suicide ideation over the previous week and experiences of suicide attempts over past year or across lifetime. All items of psychometric measures were significantly correlated in moderate degrees with the CIAS-R scores; significant inter-item correlations were also salient (S2 Table). The neuroticism score had the highest correlation coefficient (.41), followed by life impairments (.40), BSRS-5 scores (.35), inferiority (.33), depression (.32), anxiety (.31), internet use time (.29), age (-.22) and suicide ideation over the past week (.17). Multiple regression analyses with different models shown in S3 Table revealed that age, gender, neuroticism, life impairments, internet use time, and BSRS-5 explained 31% of variances for CIAS-R score. The findings indicated that CIAS-R score was mainly contributed by personality traits (neuroticism), life impairment, amount of time spent online, age (15–24) and psychopathology. Moreover, path analysis of structural equation model with Delta method was performed to explore the structural relationships among these significant risk factors and IA with a satisfactory goodness of fit (Fig A in S1 Fig). It is evident that neuroticism played the most important role to influence directly on the CIAS-R score or indirectly through the effects on the intermediate variables of internet use time, life impairment, and psychological distress. All the direct or indirect paths expressed in Fig A in S1 Fig were statistically significant at the level of p< .01.

Concerning the effects of individual items of NS (S3 Table), 6 items including problems with concentration and attention, sleeping trouble and moodiness (feeling restless, guilty, or mood swing) accounted for 20% of variances of CIAS-R score. Furthermore, with respect to the effects of BSRS-5 items, regression analysis results revealed that inferiority, depression and anxiety explained 13.2% of variances of CIAS-R.

As to the prevalence of IA based on the optimal cutoff for CIAS-R, the rate of IA was estimated as 10.6% (117/1100) (95% C.I. = 8.7–12.5%) among the study subjects. As S4 Table showed, the following personal characteristics were significantly associated with prevalence of IA: N score (High: low = 27.5%: 4.2%; OR = 8.78), life impairment (yes: no = 15.9%: 2.6%; OR = 7.31), presence of psychiatric morbidity (yes: no = 19.2%: 5.8%; OR = 3.86), younger age (15–24: 25–44: 45–80 = 19.5%: 9.8%: 6.2%; OR = 3.66: 1.64:1), male gender (males: female = 19.9%: 9.1%; OR = 2.47),and online gaming (gaming: non-gaming = 14.2%: 9.5%; OR = 1.58). With reference to DSM-5 defined severity (i.e., life impairment) of internet gaming disorder, most of individuals with IA were in mild degree (65.0%) with 26.5% in moderate degree and 8.5% in severe degree. Thus, the prevalence rate of IA with moderate and severe degree of severity was 3.7%. Compared to those without IA, the subjects with IA suffered from a significantly higher proportion of life impairment (90.6% vs. 56.9%, p < .001).

Considering psychiatric morbidity and suicide among the subjects (S4 Table), in comparison to those without IA, the subjects with IA presented with significantly higher rates of psychiatric morbidity (65.0% vs. 32.5%), suicide ideation in previous week (47.0% vs. 22.1%), suicide attempt in previous year (5.1% vs. 2.3%), and suicide attempts in lifetime (23.1% vs. 14.1%). Accordingly, as shown in S1 Table, the individuals who had suicide ideation in the past week or suicide attempt in lifetime scored significantly higher on the CIAS-R than the countered parts. Concerning the effects of all associated factors with IA, logistic regression revealed only neuroticism, life impairment and internet use time were significant predictors on IA (S5 Table).

## Discussion

Although the conceptions and definitions of IA differ in the literature, the associated psychosocial determinants found in this study were consistent with other studies. A few demographic factors were identified to have close relationship with IA. First, gender was frequently-mentioned risk factors of IA; significantly more males were identified as internet addicts than females [2–4, 12]. One of the reasons reported elsewhere was that males are more likely to play online gaming or gambling, engage in cybersex, and watch cyberporn; all of which were considered as highly associated with addictive internet use. Second, adolescents and young adults were more likely to become internet addicts as compared to other age groups [3]. It was suggested that adolescents are in the process of psychological development, thus less self-regulative, more susceptible to media influence and vulnerable to developing addictive behaviors. Third, students were more likely to be identified as IA in this study. Other than the acceptable internet use for school or job tasks, researchers indicated that college students with an insecure attachment are vulnerable to develop IA [3–12]. Specifically, the internet provides a certain psychological distance from the persons with whom insecurely attached students interact With little effort, people can make new friends and get immediate source of emotional support in the faceless cyberspace community, yet vulnerable youths are prone to addicted behavior. Thus, appropriate time for internet use and prevention of its life impairment should be emphasized among young adults, also certain key predictors of IA to be paid attention, including neurotic personality and psychological distress, as discussed below.

One of the most important psychosocial determinants for IA identified in this study was personality trait of neuroticism as assessed by the MPI- Neuroticism Scale (NS). This result was highly comparable to the findings of previous studies [2, 7, 39, 40]. Neuroticism is characterized by high levels of negative and unstable affect, such as feeling depressed, feeling restless, feeling guilty, mood swings as well as poor concentration or easy distraction. People with a higher NS score often experience negative affects when facing minor stressors. IA has been reported to closely link to stressful life events and psychological symptoms [41, 42]. Based on these characteristics, it was reasonable to infer the close association between neuroticism and IA. Our findings particularly pointed out significant associations between individual items of NS and IA, indicating that problems with concentration, distractibility during conversation, moodiness (e.g., feeling restless, mood swing and feeling guilty) and difficulty with sleep were important predictors of IA. Moreover, we noticed that co-morbidity of various psychiatric conditions with IA has been frequently reported. Our study demonstrated that individuals with IA had a significant higher rate of psychiatric morbidity (65.0%) in comparison with those without IA (32.5%), supporting by the significant correlation between CIAS-R and BSRS-5 scores. Considering the time reference of the psychometrics measured, as shown in Fig A in S1 Fig, path analysis of SEM model on IA revealed that neuroticism was the major contributing predictor. This could directly influence the severity of CIAS-R as well as indirectly contributed to the severity of psychopathology (BSRS-5 score), excessive internet use and severity of life impairment, finally contributing to the prediction of CIAS-R score.

It is evident that there are much in common for the aforementioned significant predictors for IA and the symptoms listed in the DSM5 criteria of ADHD (e.g., distractibility, difficult focusing, and restlessness) [29]. Previous reports stated a close link between IA and ADHD or ADHD symptoms [4, 6, 29]. Among the manifesting ADHD symptoms, attention deficit was reported as the most associated factors with IA [4, 6]. This study found both concentration and attention were specifically important factors related to IA severity. The neuroticism factor is a long-term stable trait; the onset of ADHD often begins before age of seven. Possibly, they share some similar mechanism to contribute to the development of IA. According to a follow-up study on ADHD, it was found that the adolescents with ADHD were more likely to develop IA in two years [43]. Another follow-up study also found that a higher proportion of the students with a higher NS score developed IA during the 2 years of follow-up in comparison to the control group [39]. These similar findings highly suggest that deficits of concentration and attention as well as moodiness or trouble with sleep were strong predictors for development of IA. Most previous reports just focused on neuroticism in general and did not mention about the roles of concentration or attention and moodiness in the development of IA, which makes one of the advantages of this study.

In terms of internet use habits, the findings showed that life impairment and increased amount of time online were important independent predictors for IA next to neuroticism. The items related to these two factors constituted a major part of CIAS-R in the subscales of "compulsive use" and "interpersonal and health problems" (12 items). Overuse of internet might also consequently create problems with time management and work together to induce negative and significant life impairment. Thus, these two factors could be causes or manifestations of IA. In addition, engagement in online gaming was particularly found to be highly correlated with the presence of IA comparing to other types of online activities like social networking. Therefore, our findings supported the concept that IA should focus on specific online activities engaged by internet users, instead of internet per se. For example, the term “internet gaming disorder” proposed by the APA in DSM-5 clearly points out “online gaming” as the media of the disordered behavior [29].

More recently, the issue of suicide among internet addicts attracted much attention in the research fields [44–47]. In this study, subjects with IA were more likely to have suicide ideation and attempts. Given that IA has been found to be highly associated with neurotic traits, psychiatric morbidity, and life impairments, all of these factors could increase suicide risks. High rates of psychiatric morbidity and suicide risks among the subjects with IA indicated that earlier identification of high risk individuals and provision of timely and pertinent management are necessary for suicide prevention and mental health promotion for the internet users. Results in this respect provide the basis for interested researchers to investigate its mechanisms and prevention for suicide behaviors among internet addicts.

In summary, the estimated prevalence of IA (10.6%) in this study is close to the worldwide reported online survey data (4–10%) for all-age internet users [48]. Our findings revealed the prevalence and severity of IA were significantly associated with several psychosocial determinants. We postulated that neurotic personality was a major predisposing factor, reacting to stressful life events as a precipitating factor to cause psychological distress. Under triggers of life events, such personality could eventually cause maladaptive coping behaviors through excessive engagement in specific internet activities, which resulting in internet addiction given significant life impairment. All these factors interacted together and progressed to internet addiction.

In this online survey of internet use and health, the findings should be interpreted under several limitations. Firstly, the female predominance of this sample may affect result interpretations and limit its generalization to female online users. However, this feature also supports recent findings that reveal the gender differences in online health information searching behaviors [49], reflecting the strong female social motive for health-related online activities in the general public. So the female-dominant sample should not be a serious problem to this study. Secondly, there were more middle-to-old-aged general public rather than youth participants in this survey, limiting the comparability between ours and previous ones; however, this can also be the advantage of this study due to its unique age constitution. Our findings filled the gap beyond prior findings focusing on IA in young people. Thirdly, generalization of the findings is confined to frequent internet users or those who are interested in the research topic because we did not have control groups of those non-responders to this online questionnaire or comparable participants for comparison. Last but not the least, causal effects between the study factors and internet addiction may not be confirmed based on our findings due to cross-sectional study design. Future studies are suggested to combine solid and acceptable diagnostic criteria as well as longitudinal design to make comprehensive and integrative formulation to the development of IA treatment and preventive strategies.

## Supporting Information

S1 Dataset Dataset for the study file named, “20150610_Internet Addiction dataset.sav” presents all study variables with anonymous data set up on 2015.06.10 for statistical analysis. (SAV)

S1 Fig Fig A of the study. (DOCX)

S1 Table Table S1 of the study. (DOCX)

S2 Table Table S2 of the study. (DOCX)

S3 Table Table S3 of the study. (DOCX)

S4 Table Table S4 of the study. (DOCX)

S5 Table Table S5 of the study. (DOCX)
