# Supplementary material for: Risk Factors of Internet Addiction among Internet Users: An Online Questionnaire Survey
Source: PLoS One. 2015 Oct 13;10(10):e0137506. doi: 10.1371/journal.pone.0137506 (PMC4603790; doi:10.1371/journal.pone.0137506)
Supplement: S1 Fig — (DOCX) [file pone.0137506.s002.docx]

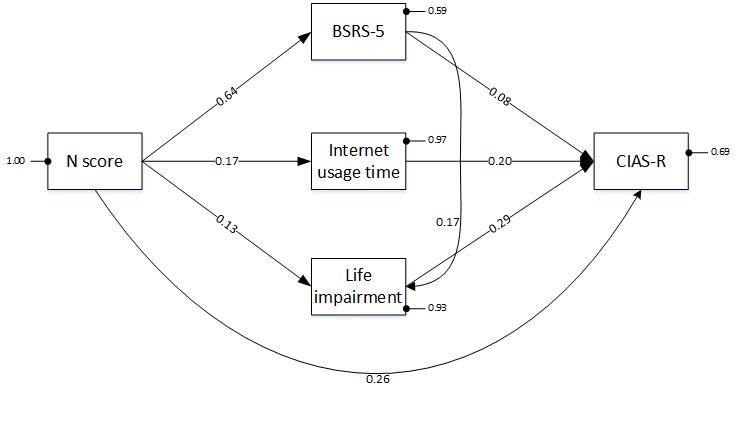


**Internet use time**

Figure 1. The path analysis by structural equation model for CIAS-R (Chi-square=16.90, d.f. =2, p =.0002, root mean square error of approximation (RMSEA) =.082, non-normed fit index (NNFI) =.933, root mean square residual (RMR) =.0313, adjusted goodness of fit index (AGFI) =.955, critical N (CN) =422.65)
