# Supplementary material for: Risk Factors of Internet Addiction among Internet Users: An Online Questionnaire Survey
Source: PLoS One. 2015 Oct 13;10(10):e0137506. doi: 10.1371/journal.pone.0137506 (PMC4603790; doi:10.1371/journal.pone.0137506)
Supplement: S1 Table — (DOCX) [file pone.0137506.s003.docx]

Table 1. Demographics and psychosocial correlates of internet addiction assessed by CIAS-R (N=1100)

| Variables | n (%) | Mean ± SD | p-value | Effect size |
| --- | --- | --- | --- | --- |
| Gender |  |  |  |  |
| Male | 156 (14.2) | 52.18 ± 16.50 | .04 | 0.20 |
| Female | 944 (85.8) | 49.23 ± 14.20 |  |  |
| Age |  |  |  |  |
| 15-24 | 164 (14.9) | 54.88 ± 14.54 | <.001^†^ | 0.06^††^ |
| 25-44 | 743 (67.5) | 50.06 ± 14.07 |  |  |
| 45 and above | 193 (17.5) | 43.65 ± 14.52 |  |  |
| Marital status |  |  |  |  |
| Single | 538 (48.9) | 53.17 ± 14.24 | <.001^†^ | 0.06^††^ |
| Married | 522 (47.5) | 46.56 ± 14.17 |  |  |
| Others | 40 (3.6) | 42.63 ± 12.84 |  |  |
| Occupation |  |  |  |  |
| Student | 126 (11.5) | 56.98 ± 12.83 | <.001^†^ | 0.04^††^ |
| Employed | 864 (78.5) | 48.35 ± 14.42 |  |  |
| Other | 110 (10.0) | 51.45 ± 15.01 |  |  |
| Online gaming |  |  |  |  |
| Yes | 260 (23.6) | 52.86 ± 14.595 | <.001 | 0.29 |
| No | 840 (76.4) | 48.66 ± 14.433 |  |  |
| Internet use time |  |  |  |  |
| < 13 hr(s) | 783 (71.2) | 47.32 ± 14.27 | <.001 | 0.57 |
| ≧13 hrs | 317 (28.8) | 55.39 ± 13.72 |  |  |
| Life impairment |  |  |  |  |
| Yes | 665 (60.5) | 54.28 ± 13.90 | <.001 | 0.87 |
| No | 435 (39.5) | 42.57 ± 12.61 |  |  |
| N-score of MPI |  |  |  |  |
| < 16 | 795 (72.3) | 46.49 ± 13.148 | .171 | 0.83 |
| ≧16 | 305 (27.7) | 57.88 ± 14.912 |  |  |
| BSRS-5 score |  |  |  |  |
| 0-5 | 705 (64.1) | 46.62 ± 13.36 | <.001 | 0.60 |
| 6-20 | 395 (35.9) | 55.06 ± 15.09 |  |  |
| Suicidal thoughts in a week |  |  |  |  |
| Yes | 272 (24.7) | 54.43 ± 15.73 | <.001 | 0.44 |
| No | 828 (75.3) | 48.08 ± 13.83 |  |  |
| Attempted suicide in the lifetime |  |  |  |  |
| Yes | 166 (15.1) | 52.68 ± 15.42 | .004 | 0.25 |
| No | 934 (84.9) | 49.11 ± 14.36 |  |  |
| Attempted suicide in the past year |  |  |  |  |
| Yes | 29 (2.6) | 54.38 ± 18.43 | .08 | 0.33 |
| No | 1071 (97.4) | 49.52 ± 14.45 |  |  |

*Mean score of CIAS-R for total subjects=49.65 ± 14.57.

^†^The analysis was performed with ANOVA; the rest was performed with t-test.

^††^The value was eta square; the rest was Cohen’s d.

BSRS-5: Five-item Brief Symptoms Rating Scale; CIAS-R: Chen Internet Addiction Scale- Revised; MPI: Maudsley Personality Inventory.
