# Supplementary material for: Risk Factors of Internet Addiction among Internet Users: An Online Questionnaire Survey
Source: PLoS One. 2015 Oct 13;10(10):e0137506. doi: 10.1371/journal.pone.0137506 (PMC4603790; doi:10.1371/journal.pone.0137506)
Supplement: S2 Table — (DOCX) [file pone.0137506.s004.docx]

Table 2. Correlation matrix of CIAS-R, BSRS-5, MPI-neuroticism, and internet use time (hours per week) by Pearson’s correlation

|  | 1. | 2. | 3. | 4. | 5. | 6. | 7. | 8. | 9. | 10. | 11. | 12. | 13. |
| --- | --- | --- | --- | --- | --- | --- | --- | --- | --- | --- | --- | --- | --- |
| 1. CIAS-R | – |  |  |  |  |  |  |  |  |  |  |  |  |
| 1. Age | -.22** | – |  |  |  |  |  |  |  |  |  |  |  |
| 1. BSRS-5 | .35** | -.16** | – |  |  |  |  |  |  |  |  |  |  |
| 1. Insomnia | .22** | .02 | .73** | – |  |  |  |  |  |  |  |  |  |
| 1. Anxiety | .31** | -.15** | .87** | .60** | – |  |  |  |  |  |  |  |  |
| 1. Hostility | .29** | -.16** | .87** | .52** | .70** | – |  |  |  |  |  |  |  |
| 1. Depression | .32** | -.18** | .88** | .52** | .70** | .76** | – |  |  |  |  |  |  |
| 1. Inferiority | .33** | -.23** | .82** | .43** | .63** | .64** | .69** | – |  |  |  |  |  |
| 1. Suicidal thoughts | .20** | -.12** | .59** | .30** | .37** | .40** | .50** | .42** | – |  |  |  |  |
| 1. MPI-Social desirability | -.17** | .21** | -.19** | -.09** | -.16** | -.17** | -.15** | -.23** | -.10** | – |  |  |  |
| 1. MPI-Neuroticism | .41** | -.24** | .64** | .40** | .53** | .55** | .63** | .55** | .40** | -.27** | – |  |  |
| 1. Internet use time (hour/week) | .29** | -.16** | .18** | .08** | .15** | .13** | .16** | .21** | .12** | -.11** | .18** | – |  |
| 1. Life impairment | .40** | -.13** | .25** | .12** | .23** | .21** | .21** | .27** | .15** | .16** | .24** | .13** | – |

Note:  ^*^p<.05, ^**^p<.01, ^***^p<.001
