# Supplementary material for: Risk Factors of Internet Addiction among Internet Users: An Online Questionnaire Survey
Source: PLoS One. 2015 Oct 13;10(10):e0137506. doi: 10.1371/journal.pone.0137506 (PMC4603790; doi:10.1371/journal.pone.0137506)
Supplement: S3 Table — (DOCX) [file pone.0137506.s005.docx]

Table 3. Models of multiple regressions (by backward elimination procedure) on internet addiction by demographics, personality, psychopathology and internet use habits

| Covariates by steps | β | SE | Adjusted R^2^ | p-value |
| --- | --- | --- | --- | --- |
| Model 1. Demographics, internet use habits and BSRS-5 score | | |  |  |
| 1. Age | -.69 | .19 | .31 | <.001 |
| 2. Gender | -1.11 | 1.06 |  | .29 |
| 3. Neuroticism | .44 | .06 |  | <.001 |
| 4. Life impairment | 5.51 | .51 |  | <.001 |
| 5. Internet use time (hour/week) | .19 | .03 |  | <.001 |
| 6. BSRS-5 | .27 | .11 |  | .01 |
| Model 2. Demographics and neuroticism scale items | |  |  |  |
| 1. Age | -0.94 | 0.20 | .22 | <.001 |
| 2. Gender | -2.40 | 1.12 |  | .03 |
| 3.Problem with concentration | 3.29 | 0.54 |  | <.001 |
| 4.Feeling restless | 2.04 | 0.60 |  | <.001 |
| 5. Difficulty with sleep | 1.47 | 0.50 |  | .004 |
| 6. Mind wanders during conversation | 1.19 | 0.50 |  | .02 |
| 7.Mood swing | 0.95 | 0.50 |  | .06 |
| 8. Feeling guilty | 1.00 | 0.56 |  | .07 |
| Model 3. Demographics and BSRS-5 items |  |  |  |  |
| 1. Age | -1.25 | 0.20 | .16 | <.001 |
| 2. Gender | -2.45 | 1.16 |  | .03 |
| 3. Inferiority | 1.80 | 0.53 |  | <.001 |
| 4. Anxiety | 1.76 | 0.62 |  | .005 |
| 5. Depression | 1.62 | 0.62 |  | .01 |
