# Supplementary material for: Risk Factors of Internet Addiction among Internet Users: An Online Questionnaire Survey
Source: PLoS One. 2015 Oct 13;10(10):e0137506. doi: 10.1371/journal.pone.0137506 (PMC4603790; doi:10.1371/journal.pone.0137506)
Supplement: S4 Table — (DOCX) [file pone.0137506.s006.docx]

Table 4. Univariate logistic regression analysis on internet addiction by demographics, internet use habits, neuroticism and psychopathology

| Covariates | Internet addiction | | | β | p-value | OR | 95% CI of OR | |
| --- | --- | --- | --- | --- | --- | --- | --- | --- |
|  | Absence  n (%) | | Presence  n (%) |  |  |  | Lower | Upper |
| Gender |  | |  |  |  |  |  |  |
| Female | 125 (12.7) | | 31 (26.5) | .91 | <.001 | 2.47 | 1.58 | 3.89 |
| Male | 858 (87.3) | | 86 (73.5) |  |  |  |  |  |
| Age |  | |  |  |  |  |  |  |
| 15-24 | 132 (13.4) | | 32 (27.4) | 1.30 | <.001 | 3.66 | 1.82 | 7.37 |
| 25-44 | 670 (68.2) | | 73 (62.4) | .50 | .124 | 1.64 | .87 | 3.09 |
| 45 and above | 81 (8.2) | | 12 (10.3) |  | <.001 |  |  |  |
| Marital status |  | |  |  |  |  |  |  |
| Married | 487 (49.5) | | 35 (29.9) |  | <.001 |  |  |  |
| Single | 457 (46.5) | | 81 (69.2) | .90 | <.001 | 2.47 | 1.63 | 3.74 |
| Other | 39 (4.0) | | 1 (0.9) | -1.03 | .316 | .36 | .05 | 2.67 |
| Occupation |  | |  |  |  |  |  |  |
| Employed | 786 (80.0) | | 78 (66.7) |  | .001 |  |  |  |
| Student | 101 (10.3) | | 25 (21.4) | .91 | <.001 | 2.49 | 1.52 | 4.10 |
| Other | 96 (9.8) | | 14 (12.0) | .39 | .214 | 1.47 | .80 | 2.70 |
| Online gaming |  | |  |  |  |  |  |  |
| Yes | 223 (22.7) | | 37 (31.6) | .46 | .033 | 1.58 | 1.04 | 2.39 |
| No | 760 (77.3) | | 80 (68.4) |  |  |  |  |  |
| Internet use time |  | |  |  |  |  |  |  |
| < 13 hr(s) | 722 (73.4) | | 61 (52.1) | .93 | <.001 | 2.54 | 1.72 | 3.75 |
| ≧13 hrs | 261 (26.6) | | 56 (47.9) |  |  |  |  |  |
| Life impairment |  | |  |  |  |  |  |  |
| Yes | 559 (56.9) | | 106 (90.6) | 1.99 | <.001 | 7.31 | 3.88 | 13.77 |
| No | 424 (43.1) | | 11 (9.4) |  |  |  |  |  |
| N-score |  | |  |  |  |  |  |  |
| < 16 | 762 (77.5) | | 33 (28.2) | 2.17 | <.001 | 8.78 | 5.71 | 13.49 |
| ≧16 | 221 (22.5) | | 84 (71.8) |  |  |  |  |  |
| BSRS-5 score |  | |  |  |  |  |  |  |
| 0-5 | 664 (67.5) | | 41 (35.0) | 1.35 | <.001 | 3.86 | 2.58 | 5.77 |
| 6-20 | 319 (32.5) | | 76 (65.0) |  |  |  |  |  |
| Suicidal thoughts |  | |  |  |  |  |  |  |
| Yes | 217 (22.1) | | 55 (47.0) | 1.14 | <.001 | 3.13 | 2.11 | 4.64 |
| No | 766 (77.9) | | 62 (53.0) |  |  |  |  |  |
| Attempted suicide in the lifetime | | |  |  |  |  |  |  |
| Yes | | 139 (14.1) | 27 (23.1) | .60 | .012 | 1.82 | 1.14 | 2.90 |
| No | | 844 (85.9) | 90 (76.9) |  |  |  |  |  |
| Attempted suicide in the past year | | |  |  |  |  |  |  |
| Yes | | 23 (2.3) | 6 (5.1) | .81 | .083 | 2.26 | .90 | 5.66 |
| No | | 960 (97.7) | 111 (94.9) |  |  |  |  |  |
