# Supplementary material for: Risk Factors of Internet Addiction among Internet Users: An Online Questionnaire Survey
Source: PLoS One. 2015 Oct 13;10(10):e0137506. doi: 10.1371/journal.pone.0137506 (PMC4603790; doi:10.1371/journal.pone.0137506)
Supplement: S5 Table — (DOCX) [file pone.0137506.s007.docx]

Table 5. Multiple logistic regression (by backward elimination procedure) on internet addiction by psychometric measures and internet use habits, adjusted by age

| Covariates | β | p-value | OR | 95% CI of OR | |
| --- | --- | --- | --- | --- | --- |
|  |  |  |  | Lower | Upper |
| 1. Age | -.22 | .25 | .80 | .55 | 1.17 |
| 2. Gender | -.85 | .001 | .43 | .26 | .72 |
| 3. Neuroticism | 1.85 | <.001 | 6.39 | 4.07 | 10.03 |
| 4. Life impairment | 1.52 | <.001 | 4.55 | 2.36 | 8.77 |
| 5. Internet use time | .56 | .01 | 1.75 | 1.13 | 2.69 |
